# Corticosteroid use in viral pneumonia: experience so far and the dexamethasone breakthrough in coronavirus disease-2019

**DOI:** 10.2217/cer-2020-0146

**Published:** 2020-11-27

**Authors:** Mohamed Rafiullah, Khalid Siddiqui

**Affiliations:** Strategic Center for Diabetes Research, College of Medicine, King Saud University, Riyadh, Saudi Arabia

**Keywords:** acute respiratory distress syndrome, ARDS, coronavirus, corticosteroids, COVID-19, dexamethasone, pneumonia

## Abstract

Dexamethasone was shown to decrease the mortality in coronavirus disease-2019 (COVID-19) recently. Use of corticosteroids was harmful in other coronavirus infections previously. WHO recommended against routine use of corticosteroids in COVID-19. In view of these, we reviewed the evidence about the use of corticosteroids in virus-induced acute respiratory distress syndrome (ARDS). Corticosteroids are beneficial in ARDS regardless of etiology. However, they increased the mortality rate in influenza-associated ARDS. In SARS and the Middle East respiratory syndrome, corticosteroids increased the mortality, delayed the viral clearance and increased the length of hospital stay. In the case of COVID-19, the available evidence from retrospective and observational studies is inconclusive about the corticosteroid use. Low-dose therapies appear to be effective. Evidence from a randomized control study found dexamethasone is effective in decreasing mortality in severe COVID-19 cases. More studies are needed to validate the benefit of corticosteroids in COVID-19.

The novel coronavirus (CoV)-2019 pandemic has become a huge public health challenge. The virus was identified as SARS CoV-2, and the disease caused by this virus is named CoV disease-2019 (COVID-19). Even though most patients are asymptomatic, in many patients, COVID-19 manifests into pneumonia, acute respiratory distress syndrome (ARDS), septic shock and death [1,2]. No drug treatment has been approved by the US FDA except remdesivir, which is given emergency use authorization. Many antivirals, convalescent plasma, immunomodulators and repurposed drugs are currently undergoing investigation. Some of the antiviral medications have already been approved in different countries. But, there is no drug available to treat the severe manifestation of ARDS in COVID-19 cases. The risk of severity and mortality of COVID-19 ranged from 12.6–23.5% and 2–4.4%, respectively. ARDS has been the most common complication of severe COVID-19 cases, with incidence ranged from 15.6 to 31% [3]. As it is one of the leading causes of death in COVID-19 patients, treating the ARDS is crucial in the recovery of COVID-19 cases.

Corticosteroids have been used to treat lung inflammation. Animal studies indicate that the use of corticosteroids improved the outcomes in viral infections [4,5]. Consequently, corticosteroids were used to manage lung inflammation during the outbreaks of the SARS and the Middle East respiratory syndrome (MERS) [6,7]. Many studies have reported no evidence of the benefit of using corticosteroids in the treatment of viral-induced lung inflammation [6,8,9]. Some studies even reported that corticosteroids’ use led to delayed viral clearance [10] and adverse outcomes [11]. WHO, in the interim guidelines on clinical management of COVID-19, released on 27 May 2020, recommended against using corticosteroids in COVID-19 cases unless indicated for other reasons [12].

Recently, dexamethasone, a synthetic corticosteroid, was shown to decrease the mortality in COVID-19 patients who were on ventilators or oxygen therapy. It is the only treatment to reduce the mortality in COVID-19 induced ARDS so far. Subsequently, many countries adopted the use of dexamethasone in critically ill patients. However, given the inconclusive nature of the evidence about the use of corticosteroids in CoV-induced ARDS, many scholars are still unconvinced about the corticosteroid treatment in COVID-19 patients. Corticosteroids induce hyperglycemia in patients with diabetes. It assumes significance as diabetes has been one of the most common comorbidities to be implicated with the risk of severe COVID-19 and death. Treating these patients with dexamethasone could deteriorate the condition. In light of these conflicting standpoints, we decided to review the evidence about the use of the corticosteroid in virus-induced ARDS.

## Corticosteroids in inflammation & ARDS

Inflammation is the result of the normal protective action of the immune system. Anti-inflammatory drugs interfere with the production and or release of inflammatory mediators. Immunosuppressant drugs suppress the immune system. Corticosteroids are both anti-inflammatory and immunosuppressant. They bind to the intracellular receptors to modulate gene transcription of pro-inflammatory mediators. Corticosteroids also inhibit the genes expressing the enzyme COX-2 to suppress the synthesis of prostaglandins, lipoxins, leukotrienes and thromboxane [13]. Corticosteroids have been used for a wide range of conditions. Systemic and inhaled corticosteroids are used to treat many respiratory disorders such as bronchial asthma, chronic pulmonary obstructive disease and pneumonitis.

ARDS is an inflammatory condition caused partly by the immune response to any lung insult. In COVID-19 patients with impaired immunity, there is massive uncontrolled viral replication and tissue damage in organs where ACE2 is expressed. Damaged tissues trigger an enormous amount of inflammatory cytokines called cytokine storm, leading to severe inflammation and pulmonary injury in COVID-19 [14]. The anti-inflammatory and immunosuppressant effect of corticosteroids would theoretically benefit patients with ARDS. Corticosteroids could dampen the cytokine storm and function as a bridge for initiating specific antiviral or disease-modifying treatments [15]. Corticosteroids are beneficial in ARDS regardless of etiology. Recent meta-analyses that compared the mortality rates among patients with ARDS concluded that corticosteroid treatment decreased the mortality rates [16–19]. Corticosteroids decreased mortality without any increase in adverse events when compared with standard therapy [16]. Corticosteroid treatment increased the number of days-off from mechanical ventilation [17,20]. When given early, low-dose corticosteroids were found to be effective in lowering the mortality [18,20]. Even though the new infection rates among patients on corticosteroid did not increase [18,20], the risk of hyperglycemia was found to be higher with corticosteroid treatment [17]. High-dose, short-term and late initiation, and preventive application of corticosteroids did not improve the mortality rate [19].

## Corticosteroids in the treatment of influenza-associated ARDS

Corticosteroids are found to increase the mortality rate in patients with influenza-associated ARDS. All the recent meta-analyses have indicated increased mortality after the use of corticosteroids [21–24]. Notably, all the meta-analyses found a significant increase in secondary infections in patients who were on corticosteroids. Patients on corticosteroid treatment were more likely to have secondary infections such as bacterial infection, invasive fungal infection or exacerbation of pre-existing conditions [22]. The high mortality rate from influenza has often been attributed to secondary bacterial infections [25]. Hence the increased mortality seen among patients receiving corticosteroids could partly be attributed to the secondary infections. Besides, treatment with corticosteroids increased the duration of intensive care unit (ICU) stay [22,23]. The duration of mechanical ventilation was not different from the control group in one meta-analysis [22], whereas it was found to be longer in another meta-analysis [23].

Treating the ARDS of viral etiology with immunosuppressant corticosteroids has always been contentious. Corticosteroid therapy prolongs the viral shedding and delays viral clearance. A high mortality rate with corticosteroid has been attributed to the prolonged viral shedding and delayed viral clearance [26]. Corticosteroids have benefitted patients who suffer from community acquired-pneumonia, as these patients will be given appropriate antibiotic therapy. On the other hand, the effect of antiviral therapy in patients undergoing corticosteroid treatment is unclear. The role of antiviral therapy has not been explored, possibly due to the lack of data. As most of the patients from the studies included in the meta-analyses were on antiviral treatment, the effect of antiviral therapy on corticosteroid-induced prolongation of viral shedding or delayed viral clearance appears to be negligible. It may be noted that most of the studies included in the meta-analyses were observational. As corticosteroids are more likely to be used in severe cases, the increased mortality may be associated with the disease’s severity.

## Corticosteroids in the treatment of previous CoV diseases

SARS and MERS are previous CoV infections that were reported in 2003 and 2012. Symptoms and viral phylogenetics of these infections resemble COVID-19. A systematic review of treatment effects in SARS patients reported that out of 29 studies that included corticosteroid treatment, 25 were inconclusive, and four possibly caused harm [7]. Thirteen studies used ribavirin antiviral therapy. Delayed viral clearance, the onset of diabetes, avascular necrosis and osteoporosis were the possible harm caused by the corticosteroid treatment. In a meta-analysis of corticosteroid therapy on CoV infections, subgroup analysis of SARS patients indicated that corticosteroids did not decrease the mortality when compared with the controls, but, delayed the viral clearance and increased the length of hospital stay in SARS and MERS patients [27].

In another meta-analysis, neither SARS nor MERS patients had a higher mortality rate after corticosteroid treatment, though, the overall analysis indicated an increase in mortality with corticosteroid treatment. Corticosteroid also increased the length of hospital stay in both SARS and MERS patients. Patients treated with corticosteroids were more likely to experience adverse events. Increased bacterial infections and hypokalemia were reported more in patients who were on corticosteroids [28]. In one of the randomized, double-blind, placebo-controlled studies, early use of hydrocortisone (<7 days of illness) resulted in a higher viral load in the second and third week of illness in SARS patients. The study also found delayed viral clearance in patients who were on corticosteroid (median: 12 days [11–20 days] vs 8 days [8–15 days]) [29]. In a study on critically ill MERS patients, corticosteroid therapy did not result in increased mortality. However, it was associated with delayed viral clearance [6].

## Corticosteroids in the treatment of COVID-19

Prudent use of corticosteroids has been reported in patients with COVID-19 [2]. Several studies have reported the effect of corticosteroids in COVID-19 (Table 1). A systematic review and meta-analysis evaluated clinical features and treatment outcomes of COVID-19 from all published studies up to 15 March 2020. Pooled data from 16 studies with 2407 patients reported the use of corticosteroids. The rate of mortality and ARDS were 7.2% (95% CI: 1.7–15.4) and 22.7% (95% CI: 9.9–38.6), whereas the overall rates of mortality and ARDS were 4.3 and 18.4%, respectively. Meta-regression analysis of the data revealed an association between corticosteroid use and ARDS [30]. Another systematic review that collected publications until 15 March 2020 evaluated the use of corticosteroids in COVID-19. The review does not fully support the routine use of corticosteroids in COVID-19. Out of the four studies included in the analysis, two reported possible harm by the use of corticosteroids (increased risk of ICU admissions [72.2 vs 35.3%] and delayed viral clearance [15 vs 8 days]). Meanwhile, in another study, methylprednisolone significantly decreased the risk of mortality by 62% in patients with severe pneumonia (hazard ratio: 0.38; 95% CI: 0.20–0.72) [31].

**Table 1.** Effect of corticosteroids in coronavirus disease-2019.

Study (year)	Type of study	Control	Treatment	Number of patients	Outcome	Ref.
Zhang *et al.* (2020)	Systematic review and meta-analysis	Standard care	Different available treatments	45 studies with 4203 patients	Corticosteroids were associated with a higher rate of ARDS	[30]
Veronese *et al.* (2020)	Systematic review	Standard care	Methylprednisolone	Four studies with 542 Chinese patients	From four studies, each reported a different outcome • Intravenous methylprednisolone: no benefit • Greater risk of ICU admission (72.2 vs 35.3%, p < 0.001) • Methylprednisolone decreased mortality, HR: 0.38 (0.20–0.72) • Delayed viral clearance: corticosteroids vs control oropharyngeal swabs: 15 vs 8 days Feces: 20 vs 11 days	[31]
Zhong *et al.* (2020)	Meta-analysis	Standard care	Different available treatments	18 studies with 4941 patients (SARS, MERS and COVID-19)	A combination of ribavirin and corticosteroids decreased mortality (RR: 0.43, 95% CI: 0.27–0.68)	[32]
Fadel *et al.* (2020)	Multicenter quasi-experimental study	Standard care	Low-dose iv. methylprednisolone 0.5–1 mg/kg/day two divided doses for 3 days	231 patients (81/132)	• Mortality: 0.45 (0.22–0.91) • Respiratory failure requiring mechanical ventilation: 0.47 (0.25–0.92) • Escalation to ICU: 0.47 (0.25–0.88)	[33]
Zha *et al.* (2020)	Observational study	Standard care	Corticosteroids	31 patients	• Virus clearance time: HR: 1.26 (0.58–2.74) • Length of hospital stay: 0.77 (0.33–1.78) • Duration of symptoms: 0.86 (0.40–1.83)	[34]
Gong *et al.* (2020)	Retrospective study	Standard care	Methylprednisolone	34 patients under 50 years age	Relieved the symptoms • Persistent fever • Difficult breathing • Improve oxygenation Prevented disease progression	[35]
Fang *et al.* (2020)	Retrospective analysis	Standard care	Low-dose methylprednisolone	78 patients	No difference in time to viral clearance	[36]
Selvaraj *et al.* (2020)	Case series	Nil	Dexamethasone	21 patients	78% of patients discharged Length of hospital stay: 7.8 days	[37]
Li *et al.* (2020)	Retrospective analysis	Standard care	Methylprednisolone	206 patients	High dose (80 mg/day) delayed viral shedding of patients	[38]
Yuan *et al.* (2020)	Retrospective cohort study based on propensity-score matched analysis	Standard care	Methylprednisolone	70 patients with non-severe COVID-19 pneumonia	Corticosteroid group, more patients progressed to severe disease 11.4 vs 2.9% (p = 0.353)	[39]
RECOVERY trial (2020)	Randomized controlled study	Standard care	Dexamethasone	4321 patients control and 2104 patients in treatment group	Mortality: • Age-adjusted RR = 0.83 (95% CI: 0.74–0.92; p < 0.001) • Patients on invasive mechanical ventilation (29.0 vs 40.7%; RR: 0.65; 95% CI: 0.51–0.82; p < 0.001) • Patients who were on oxygen (21.5 vs 25.0%; RR: 0.80; 95% CI: 0.70–0.92; p = 0.002) • Patients who did not receive respiratory support at randomization (17.0 vs 13.2%; RR: 1.22; 95% CI: 0.93–1.61; p = 0.14).	[40]

ARDS: Acute respiratory distress syndrome; COVID-19: Coronavirus disease-2019; HR: Hazard ratio; ICU: Intensive care unit; iv.: Intravenous; MERS: Middle East respiratory syndrome; RR: Rate ratio.

A meta-analysis that combined the data from all the CoV infections such as SARS, MERS and COVID-19 showed a significant reduction in mortality with a combination of ribavirin and corticosteroid in the subgroup analysis (rate ratio [RR]: 0.43; 95% CI: 0.27–0.68). It also increased viral clearance when compared with control [32]. In a multicenter quasi-experimental study, early short course of methylprednisolone (0.5–1 mg/kg/day divided in two intravenous doses for 3 days) decreased the mortality (odds ratio [OR]: 0.45; 95% CI: 0.22–0.91), respiratory failure requiring mechanical ventilation (OR: 0.47; 95% CI: 0.25–0.92) and escalation to ICU (OR: 0.47; 95% CI: 0.25–0.88). Patients on early corticosteroid treatment also reported a significant reduction in median hospital length of stay [33]. In an observational study of patients from two hospitals, corticosteroid treatment was not associated with virus clearance time, hospital length of stay and duration of symptoms. However, the study patients were younger (median 39 years, interquartile range [IQR]: 32–54) and had a mild COVID-19 without ARDS [34].

In a retrospective observational study of COVID-19 patients under 50 years of age, corticosteroid treatment improved the symptoms. Nevertheless, it delayed viral clearance in patients [35]. In another retrospective analysis, patients were divided into severe and non-severe groups to adjust for baseline data inconsistency. Patients were on low-dose methylprednisolone. Viral clearance was not delayed in patients who were on corticosteroids when compared with the controls. It was observed both in severe and non-severe groups [36]. In a case series of 21 COVID-19 patients, who were given a short course of dexamethasone, CRP levels decreased significantly at the time of discharge. None of the patients had an escalation to mechanical ventilation. The mean length of hospital stay was found to be 7.8 days [37]. Li *et al.*, 2020 found that only high-dose corticosteroid (80 mg/day methylprednisolone) delayed the viral clearance in COIVD-19 patients while the low dose did not [38]. A retrospective study-based propensity score-matched cohort found that more patients in the corticosteroid group progressed to severe COVID-19 (11.4 vs 2.9%; p = 0.353). Other parameters such as hospital stay and duration of viral shedding were prolonged, while fever time shortened, though, these changes were not statistically significant [39].

The effect of dexamethasone 6 mg/day for up to 10 days was evaluated in a randomized, controlled, open-label clinical trial that compared different treatments with usual care (RECOVERY). This is the only study to evaluate the corticosteroid treatment in COVID-19 in a randomized controlled trial [40]. Two thousand one hundred four patients were randomized to receive dexamethasone from 176 National Health Service hospitals in the United Kingdom. A total of 21.6% of patients allocated to dexamethasone and 24.6% of patients allocated to usual care died within 28 days. Age-adjusted RR was 0.83 (95% CI: 0.74–0.92; p < 0.001). Dexamethasone decreased deaths in patients who received invasive mechanical ventilation (29.0 vs 40.7%; RR: 0.65; 95% CI: 0.51–0.82; p < 0.001), or those who received oxygen (21.5 vs 25.0%; RR: 0.80; 95% CI: 0.70–0.92; p = 0.002). Notably, dexamethasone did not decrease mortality in patients who did not receive respiratory support at randomization (17.0 vs 13.2%; RR: 1.22; 95% CI: 0.93–1.61; p = 0.14). There was a slight increase in mortality with corticosteroid in patients who did not require respiratory support. Also, there was no data about viral clearance rates among patients. As corticosteroids could delay the viral clearance, it will be important to see the outcome of patients after 28 days. A total of 28% of patients were still in the hospital at the end point of the study, which is 28 days. Critics are of the concern that the trial results are from a subgroup analysis that was not prespecified [41]. There were 25% of diabetes patients among the participants in the trial. Dexamethasone use would have worsened their glycemic control. Poorly controlled diabetes resulted in higher mortality from COVID-19 [42]. Investigators must have carefully considered these facts before treating diabetes patients with dexamethasone. Even though the distribution of diabetes patients between the groups is similar in the RECOVERY trial, the mortality rate might have differed. Therefore, it is essential to see the outcome of dexamethasone treatment in patients with diabetes separately.

Dexamethasone is the only drug proven to decrease the mortality rate in COVID-19 patients. However, it is useful only in severe cases of COVID-19. In patients who do not require oxygen or mechanical ventilation, dexamethasone slightly increased mortality [40]. On the other hand, most of the other available evidence suggest corticosteroid treatment in COVID-19 has either no benefits or harmful. It led to severe disease, increased mortality and delayed viral clearance. But these data come from retrospective and observational studies, thus making it difficult to conclude. As sicker people are more likely to have received corticosteroids, higher mortality cannot be attributed to the corticosteroid treatment alone. Another concern with corticosteroid treatment is delayed viral clearance. The immunosuppressant effect of corticosteroids is possibly impairing the viral clearance by host immunity. In bacterial pneumonia, appropriate antibiotic treatment is effective in clearing the pathogens. It is not the case with the viral infection. With the not so effective antiviral therapies, immunosuppressant corticosteroids could worsen the viral clearance. Several evidences suggest that low-dose corticosteroids did not worsen the viral clearance in COVID-19. Early, short course of corticosteroid use decreased the mortality and length of hospital stay in other CoV infections. On the other hand, results from the RECOVERY trial clearly show that only severe COVID-19 patients benefited from dexamethasone treatment. While low-dose corticosteroid appears to be effective but an early application is not. Hyperglycemia is another well-known adverse effect of corticosteroid treatment. It assumes significance here because diabetes is one of the risk factors for severe complications of COVID-19 and its mortality. Surprisingly, the RECOVERY trial results did not provide data on diabetes patients who were on dexamethasone. Therefore, in the absence of any direct evidence, it would be advisable to follow the established protocols in using corticosteroids for treating the COVID-19 patients with diabetes.

## Conclusion

Corticosteroids decrease the mortality from ARDS of any etiology. However, they increased the mortality in influenza-associated ARDS. The effect of corticosteroid treatment in different CoV infections largely appears to be harmful. In COVID-19, the available literature from retrospective and observational studies is inconclusive about the corticosteroid use. The recent RECOVERY trial, the only available evidence from a randomized control study so far, shows that dexamethasone effectively decreases mortality in severe COVID-19 cases that require mechanical ventilation or oxygen therapy. Care should be exercised in treating patients with diabetes. More studies are needed to validate the benefit of corticosteroids in COVID-19.

**Executive summary**

- Corticosteroids are beneficial in acute respiratory distress syndrome (ARDS) regardless of etiology.
- When given early, low-dose corticosteroids were found to be effective in lowering the mortality and increased the number of days-off from mechanical ventilation in ARDS of any etiology.
- High-dose, short-term and late initiation, and preventive application of corticosteroids did not improve the mortality rate in ARDS of any etiology.
- Corticosteroids have benefitted patients who suffer from community acquired-pneumonia.
- Corticosteroids increase the mortality rate and duration of the intensive care unit stay in patients with influenza-associated ARDS.
- Patients on corticosteroid treatment were more likely to have secondary infections such as bacterial infection, invasive fungal infection or exacerbation of pre-existing conditions.
- Delayed viral clearance, the onset of diabetes, avascular necrosis and osteoporosis were the possible harm caused by the corticosteroid treatment in SARS and the Middle East respiratory syndrome.
- Corticosteroid also increased the length of hospital stay in both SARS and Middle East respiratory syndrome patients.
- Early use of hydrocortisone (<7 days of illness) resulted in a higher viral load in the second and third week of illness in SARS patients.
- Data from observational and retrospective studies were inconclusive about the benefit of harm from the use of corticosteroids in coronavirus disease-2019 (COVID-19).
- Early, short-course and low-dose corticosteroids did not delay the viral clearance in COVID-19 patients.
- Dexamethasone decreases mortality in severe COVID-19 cases that require mechanical ventilation or oxygen therapy.
